# Molecular Approaches for Diagnosis, Therapy and Prevention of Cow’s Milk Allergy

**DOI:** 10.3390/nu11071492

**Published:** 2019-06-29

**Authors:** Birgit Linhart, Raphaela Freidl, Olga Elisyutina, Musa Khaitov, Alexander Karaulov, Rudolf Valenta

**Affiliations:** 1 Department of Pathophysiology and Allergy Research, Medical University of Vienna, 1090 Vienna, Austria; 2 NRC Institute of Immunology FMBA of Russia, 115478 Moscow, Russia; 3 Laboratory of Immunopathology, Department of Clinical Immunology and Allergy, Sechenov First Moscow State Medical University, 119435 Moscow, Russia

**Keywords:** cow’s milk allergy, cow’s milk allergens, diagnosis of cow’s milk allergy, molecular diagnosis, treatment of cow’s milk allergy, prevention of cow’s milk allergy

## Abstract

Cow’s milk is one of the most important and basic nutrients introduced early in life in our diet but can induce IgE-associated allergy. IgE-associated allergy to cow’s milk can cause severe allergic manifestations in the gut, skin and even in the respiratory tract and may lead to life-threatening anaphylactic shock due to the stability of certain cow’s milk allergens. Here, we provide an overview about the allergen molecules in cow’s milk and the advantages of the molecular diagnosis of IgE sensitization to cow’s milk by serology. In addition, we review current strategies for prevention and treatment of cow’s milk allergy and discuss how they could be improved in the future by innovative molecular approaches that are based on defined recombinant allergens, recombinant hypoallergenic allergen derivatives and synthetic peptides.

## 1. Introduction

IgE-associated food allergy is much less common than respiratory allergy [1]. It usually affects only a small percent of the population, whereas more than 25% of the population suffers from respiratory allergy. Nevertheless, food allergy is a very important topic because the number of subjects with a perception of having food allergy is much higher than the number of patients with confirmed IgE-associated food allergy [2]. This can be attributed to the fact that there are different forms of food intolerance, of which immunologically mediated food allergy represents only a relatively small portion. IgE-associated allergy to milk and in particular to cow’s milk is one of the most important forms of food allergy because it can cause severe and life-threatening symptoms and affects children early in life [2]. In the European EuroPrevall birth cohort, 12,049 children were enrolled, of whom 9336 (77.5%) were followed up to 2 years of age. Cow’s milk allergy CMA was confirmed in 55 children by oral food challenge, resulting in an incidence of challenge-proven CMA of 0.54% [3]. The results from the EuroPrevall study thus confirm that CMA is rare and indicate that robust and simple diagnostic tests are needed to confirm and, perhaps even more important, to exclude IgE sensitization to cow’s milk allergens early in life. We have suggested the use of micro-arrayed allergens in the format of allergen chips as a possibility to test for IgE sensitization to multiple allergen molecules with small amounts of blood in children and demonstrated the feasibility of this approach in European birth cohorts in the FP7-funded European project ‘Mechanisms of the Development of Allergy’ MeDALL [4,5]. This technology is based on purified single allergen molecules which are produced by recombinant expression or purification from the natural allergen sources. Major advantages of molecular testing are that the culprit allergen molecules can be precisely identified. The latter is of particular interest for CMA because it was found that clinical phenotypes of patients with CMA may vary depending on the molecular sensitization pattern. Therefore, in the next section, we provide a short overview of the cow’s milk allergen molecules, their characteristics and how IgE recognition of these molecules may be linked to clinical phenotypes.

## 2. Cow’s Milk Allergen Molecules

**Figure 1:**
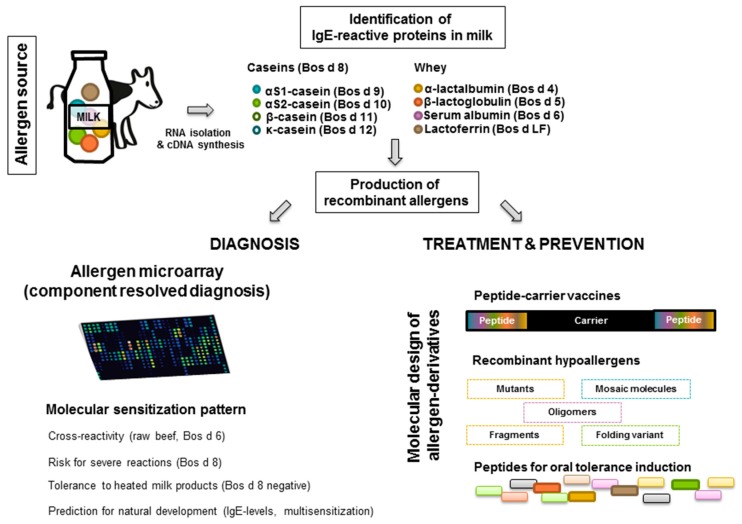
Component-resolved diagnosis, treatment, and prevention of cow’s milk allergy. Cow’s milk contains several allergenic molecules (components), which can be produced as recombinant proteins. Microarray technology allows determining reactivity profiles of patients and their sensitization to cross-reactive allergens and identifying individual allergens that cause disease. The severity of reactions and natural tolerance development can also be predicted. Based on the DNA sequences of cow’s milk allergens, molecules for treatment and prevention can be designed.

In principle, it is possible to obtain large amounts of natural cow’s milk allergens by biochemical purification from milk but the recombinant expression of allergen-encoding DNA allows obtaining highly pure allergens without contaminations with other allergens from the same allergen source (Figure 1). Accordingly, recombinant cow’s milk allergens can be obtained by expression in different host systems and used in multiallergen tests. According to the amino acid sequences of the individual cow’s milk allergens, it is possible to construct different recombinant hypoallergenic derivatives and to prepare synthetic peptides for innovative forms of treatment and prevention (Figure 1).

### 2.1. Whey Proteins

#### 2.1.1. Alpha-Lactalbumin (Bos d 4)

Alpha-lactalbumin is a small, monomeric (14.19 kDa) Ca²^+^-binding protein (Figure 1, Table 1). Besides calcium binding, four disulfide bridges stabilize the structure of the molecule [6,7,8]. 

Bovine and human alpha-lactalbumin show a high (>70%) amino acid sequence similarity [7,8]. Despite this fact, alpha-lactalbumin contains genuine, milk-specific IgE epitopes which can be explained by the observation that the IgE epitopes are clustered at the N- and the C-terminal end of the protein, which differ greatest between human and bovine alpha-lactalbumin [8,9]. Human IgE-antibodies are mainly directed to conformational epitopes, which can be possibly attributed to its high stability and structural refolding capacity to heat [6]. However, IgE-reactivity fragments generated from alpha-lactalbumin by tryptic digestion has also been demonstrated [8].

#### 2.1.2. Beta-Lactoglobulin (Bos d 5)

Beta-lactoglobulin is a small (18.3 kDa) lipocalin family protein (Figure 1, Table 1). The protein contains two disulfide bridges and one free cysteine that may cause dimerization of lactoglobulin [6,8]. The presence of disulfide bridges in the molecule is also associated with its high stability to proteolytic cleavage [7,10]. Even after digestion in the gastrointestinal tract, bovine beta-lactoglobulin is found to be secreted in human breast milk [8]. Peptides generated by the tryptic *in vitro* digestion of beta-lactoglobulin, as well as synthetic beta-lactoglobulin derived peptides, showed IgE reactivity and thus confirmed the presence of linear IgE binding epitopes in the beta-lactoglobulin amino acid sequence [11,12,13]. Therefore, the secretion of beta-lactoglobulin in breast milk could potentially cause symptoms in cow’s milk allergic infants or sensitization. No human homologous protein to bovine beta-lactoglobulin is found in breast milk. It is thought that heating from 50–90 °C increases the allergenicity of beta-lactoglobulin, as linear, usually hidden epitopes become available due to the alteration of the native conformation of the protein. In contrast heating above 90 °C is thought to mask linear, as well as conformational epitopes, as beta-lactoglobulin forms aggregates [6]. 

#### 2.1.3. Serum Albumin (Bos d 6)

Bovine serum albumin (BSA) only accounts for 5% of total whey protein content but is recognized by up to 50% of cow’ milk allergic patients [6,11] (Figure 1, Table 1). Similar to alpha-lactalbumin, BSA (67 kDa) shows a high amino acid sequence homology to its human counterpart [11]. BSA not only plays a role in cow’s milk allergy but seems to be also important in beef allergy. There is, however, evidence that cooking destroys the allergenic activity of BSA [14]. The identification of sensitization to BSA by component resolved diagnosis may therefore potentially predict whether a cow’s milk allergic infant will also suffer from allergic symptoms when beef is introduced into the diet. Since BSA shows sequence and structural similarity with albumins from respiratory allergen sources such as cats, dogs, horses and small furry animals [15,16,17], it may be important to test for cross-reactivity to confirm whether the genuinely sensitizing allergen source is milk or a respiratory allergen source.

**Table 1:** Characteristics of cow’s milk allergens

	Whey					Caseins				
Allergen (UNIPROT)	Bos d 4 (B6V3I5)	Bos d 5 (G5E5H7)	Bos d 6 (B0JYQ0)	Bos d 7	Bos d LF (B9VPZ5)	Bos d 8	Bos d 9 (B5B3R8)	Bos d 10 (P02663)	Bos d 11 (P02666)	Bos d 12 (P02668)
**Isoallergen (Accession number)**	Bos d 4.0101 (P00711)	Bos d 5.0101 (P02754), Bos d 5.0102 (B5B0D4)	Bos d 6 (P02769)	Bos d 7.0101	Bos d LF (P24627)		Bos d 9.0101 (P02662)	Bos d 10.0101 (P02663)	Bos d 11.0101 (P02666)	Bos d 12.0101 (P02668)
**Protein family**	Albumins	Globulins, Lipocalins	Albumins	Immuno-globulins	Transferrins	Caseins	Caseins	Caseins	Caseins	Caseins
**Protein name**	Alpha-lactalbumin	Beta-lactoglobulin	Serum albumin	IgG	Lactoferrin	AlphaS1-casein, alphaS2-casein, beta-casein, kappa-casein	AlphaS1-casein	AlphaS2-casein	Beta-casein	Kappa-casein
**Molecular weight [kiloDalton]**	14.19	18.31	67.20	160	76.14		22.89	24.35	23.58	18.97
**Isoelectric point**	4.80	4.83	5.60	n.a.	8.67		4.95	8.34	5.13	5.93
**Number of amino acids (AA)**	123 (AA 1-19 signal; AA 20-142 chain)	162 (AA 1-16 signal; AA 17-162 chain)	589 (AA 1-18 signal; AA 19-607)	n.a.	689 (AA v1-19 signal; AA 20-708 chain)		199 (AA 1-15 signal; AA 16-214 chain)	207 (AA 1-15 signal; AA 16-222 chain)	209 (AA 1-15 signal; AA 16-224 chain)	169 (AA 1-21 signal; AA 22-190 chain)
**Number of patients (n), %IgE-positive**	n = 58, 27.6% [18]; n = 140, 25% [19]; n = 78, 62.8% [20]; n = 51, 19.6% [21]; n = 45, 19% [22]	n = 58, 38.7% [18]; 10% [19]; n = 78, 43.6% [20]; n = 140, n = 51, 19.6% [21]; n = 45, 23% [22]	n = 58, 12.9% [18]; 25% [19]; n = 78, 3.8% [20]; n = 140; n = 51, 21.56% [21]; n = 45, 23% [22]	n = 58, 10.3% [18]; n = 140, 40% [19]	n = 58, 10.3% [18]; n = 140, 10% [19]; n = 78, 5.1% [20]; n = 51, 66.67% [21]	n = 58, 46.5% [18]; n = 140, 40% [19]; n = 51, 49.02% [21]; n = 45, 40% [22]	n = 58, 58% [18]; n = 140, 25% [19]		n = 58, 71.0% [18]; n = 140, 20% [19]	n = 58, 58.1% [18]; n = 140, 10% [19]; n = 78, 29.5% [20]
**Cross reactivity**		n = 6,beta-lactoglobulin from buffalo’s, ewe’s milk, goat’s milk [23]	raw meat (beef, lamb, deer, pork) [24]; Cap h 6 (goat), Ovi a 6 (Sheep), Equ c 3 (horse), Equ as 6 (Donkey), Sus s 1 (Pig) [6]			Casein from buffalo’s, ewe’s milk, goat’s milk [23]; 30S component from soy (IgE-reactivity) [25]				
**Heat stability**	Aggregation [26]	Aggregation [26]	Heat-sensitive	?	?	Heat-stabile				
**Associated symptoms**			Bos d 6-sensitized beef allergic children react to CM [27]; patients tolerated heated meat products [14,24]			Bos d 8-sensitized CM allergic patients react to heated products, Bos d 8-sIgE correlates with symptom severity [28]				

#### 2.1.4. Bos d 7 (Immunoglobulin)

Immunoglobulin G (IgG) has been reported to be recognized by IgE from a varying percentage (i.e.;10–40%) of cow’s milk allergic patients [18,19] (Table 1). The allergenic activity of IgG has not yet been evaluated and there is no information regarding its clinical relevance. It is possible that carbohydrate epitopes of IgG represent IgE epitopes similarly as has been reported for the cat allergens Fel d 5 (cat IgA) and Fel d 6 (cat IgM), both of which seem to have no clinical importance and allergenic activity [29,30]. A recent study revealed that the dominant IgE epitope on cat IgA2 is the carbohydrate α-gal, which exists on a large number of other mammalian proteins [31]. However, the role of carbohydrate epitopes on Bos d 7 for IgE recognition still has to be determined.

#### 2.1.5. Lactoferrin (Bos d LF)

Lactoferrin is an iron-binding glycoprotein belonging to the transferrin protein family. By chelating iron, it deprives bacteria from iron uptake and thus acts as natural antimicrobial protein in milk [7,32]. It has been reported that lactoferrin is recognized by IgE antibodies from cow’s milk allergic patients at widely varying percentages (i.e.; 5–66%) (Table 1) [18,19,20,21]. The clinical relevance of lactoferrin is not known. Lactoferrin-specific IgE antibodies have been identified in sera from cow’s milk allergic patients but its allergenic activity and the impact of lactoferrin sensitization on severity of clinical symptoms have not yet been investigated [11].

### 2.2. Caseins (Bos d 8)

#### AlphaS1-Casein, AlphaS2-Casein, Beta-Casein, Kappa-Casein (Bos d 9–12)

Caseins are calcium-binding phosphoproteins that constitute 80% of total milk protein. The high calcium content in milk enables the formation of casein micelles. Four distinct casein proteins are recognized as individual allergens (alphaS1-casein, alphaS2-casein, beta-casein, kappa-casein, Bos d 9–12) [6] (Figure 1, Table 1). Bos d 9–12 contain cross-reactive as well as non-cross-reactive IgE epitopes among each other [20]. In contrast to whey proteins, caseins are heat stable [6]. However, caseins are highly susceptible to enzymatic degradation [6]. Tryptic digest fragments of aS1-casein exerted no immunoreactivity [8,33] It might be reasonable to assume that the sensitivity to digestion is the reason why casein-specific IgE antibodies are directed towards linear epitopes [11].

The cross-reactivity of caseins is potentially explained by the conserved region containing the phosphorylation site for alpha- and beta-caseins [8]. However, the measurement of IgE reactivity to purified recombinant caseins indicated that patients’ IgE antibodies could discriminate the different casein allergens [20,33]. Moreover, natural milk allergen preparations may be contaminated with allergens from the same source, and residual casein fragments were detected in the whey fraction of cow’s milk [34].

## 3. Diagnosis of Cow’s Milk Allergy: From Classical Procedures Towards Molecular Diagnosis

Several guidelines for the diagnosis of cow’s milk allergy have been published during the recent years [2,35,36]. Recording the medical history and physical examination of the patient is one cornerstone in the diagnosis. CMA can show a variety of clinical manifestations and it is important to discriminate immune-mediated reactions from non-immune-mediated adverse reactions, like toxic, and pharmacologic reactions, or lactose intolerance the most common form of milk intolerance which results from a reduced availability of enzymes to digest lactose and causes symptoms only in the bowel [1,37]. Immune-mediated symptoms can be classified according to Coombs and Gell in type I hypersensitivity reactions mediated by IgE antibodies due to sensitization to cow’s milk allergens and type II-type IV reactions [1]. IgE-mediated cow’s milk allergy can be clearly diagnosed by demonstration of the presence of allergen-specific IgE antibodies while for non-IgE-mediated food allergy no unambiguous diagnostic tests are available. Symptoms of IgE-associated cow’s milk allergy can appear as immediate (early) reactions and delayed (late) reactions. While immediate reactions are observed within the first minutes up to 2 hours after milk consumption and represent IgE-mediated effects, delayed reactions may appear up to 48 hours or even 1 week after ingestion and may be attributed to cell-mediated immune mechanisms [36]. IgE-mediated allergen presentation to T cells is one possible mechanism involved in the late response to allergens [38] but it has also been shown that T cell-mediated allergen-specific inflammation can occur via a non-IgE-mediated mechanism [39,40]. It has to be considered that both early and late symptoms can occur in the same patient and may affect different organs. This mainly includes the gastrointestinal tract, leading to abdominal pain, vomiting, diarrhea, dysphagia, constipation, occult blood loss, iron-deficiency anemia, and the skin by developing urticaria, atopic eczema, or angioedema, and also respiratory symptoms like runny nose, wheezing, and allergic asthma, and more general and severe reactions as systemic anaphylaxis may occur, but are fortunately rare. Even acute coronary syndromes associated with anaphylactic reactions to milk have been observed [41,42,43]. IgE-mediated symptoms occur in approximately 50% of children with CMA, while they are rarer in adults, as CMA resolves in more than 85% of cases [2]. 

In contrast, non-IgE-mediated immune reactions associated with cow’s milk ingestion symptoms are often restricted to the gastrointestinal tract and the skin, they are often of the delayed type and develop up to several days after milk intake. In these patients, no circulating cow’s milk allergen-specific IgE antibodies can be detected and they have a negative skin reaction to cow’s milk proteins. These reactions include food protein-induced enterocolitis syndrome, cow’s milk protein-induced enteropathy, and cow’s milk-induced proctitis and proctocolitis [2], and also conditions where milk protein-specific IgE antibodies are present may occur, like in eosinophilic esophagitis or gastroesophageal reflux disease.

Once the medical history suggests the presence of cow’s milk allergy, a number of *in vitro* and *in vivo* diagnostic tests are performed. IgE sensitization to milk proteins is evaluated by the determination of specific IgE antibodies in serum. Skin testing includes the skin prick test (SPT) for IgE-mediated immediate allergic responses and the atopy patch test (APT) measuring T cell-mediated late responses to allergens. The APT can be useful to demonstrate the involvement of T cells in the pathogenesis. Still the double blind, placebo-controlled food challenge (DBPCFC) is considered as a gold standard for the diagnosis of milk allergy [2]. However, many clinicians avoid it because they fear severe and life-threatening side effects and the test procedure is very cumbersome, involving hospitalization and the availability of emergency care.

### 3.1. Cow’s Milk-Specific IgE Measurement

The *in vitro* determination of allergen-specific IgE levels represents an important tool for the diagnosis of IgE-mediated milk allergy because it allows the unambiguous demonstration of IgE sensitization. It is performed in serum samples obtained from patients, whereby several test systems are available like the ImmunoCAP system, the Immulite assay system, or multiplex allergy tests [44,45]. It was recently proposed to measure specific IgE in the saliva instead of blood samples, because it is difficult to obtain blood samples from young children [46]. However, IgE concentrations in saliva and other body fluids are usually lower than in blood and may vary strongly due to secretion. Therefore, multiallergen testing using chips containing micro-arrayed allergens should be preferred because it requires on few microliters of blood and serum [47].

The sensitivity and specificity of IgE measurements is very high. However, the presence of allergen-specific IgE in the blood as well as a positive skin test result does not necessarily correlate with the development of allergic symptoms [2]. Therefore, specific IgE levels have to be evaluated in the context of the clinical history of the patient. High milk allergen-specific IgE levels were shown to be predictive for the development of clinical symptoms during an oral food challenge. A number of studies analyzed the cut-off levels for milk-specific IgE for predicting the onset of milk-induced allergic symptoms [48]. However, different values were obtained due to the various study populations, and the criteria to evaluate the severity of symptoms.

In the context of serological testing, it is important to state clearly that non-IgE-mediated cow’s milk allergy cannot be diagnosed by determination of milk allergen-specific IgG and IgA antibody levels, which was shown to be inappropriate for diagnosing cow’s milk allergy [49] and is also not recommended by national and international guidelines [50,51].

### 3.2. Skin Testing

#### 3.2.1. Skin Prick Test (SPT)

The SPT represents a commonly used diagnostic method in clinical practice, as it is cheap and easy to perform. However, its diagnostic value is limited, as the specificity of this test is rather low. Several studies tried to define a cut-off value to predict CMA in the tested patients. Using commercial cow’s milk extracts, wide variations of cut-offs ranging from 4.3–20 mm were found [48,52,53,54,55,56,57]. Studies comparing cow’s milk extract to purified natural α-lactalbumin, ß-lactoglobulin, and casein indicated that fresh cow’s milk was the most sensitive SPT reagent [57]. Having a positive SPT for all three cow’s milk allergens increased the likelihood of a positive response to challenge [55]. Therefore, reagents containing defined amounts of pure allergen components would be needed for the *in vivo* diagnosis of CMA [58,59].

#### 3.2.2. Atopy Patch Test (APT)

The APT is most often used for the diagnosis of atopic dermatitis but has also been proposed for the diagnosis of non-IgE-mediated CMA and eosinophilic esophagitis [2,60]. As the APT measures cow’s milk allergen-induced T cell activation, it can only detect allergic reactions of the delayed type which may be IgE-dependent or IgE independent [39,40]. It is performed by the application of cow’s milk allergens to the skin via a patch, which is fixed for 24–48 h. After the removal of the patch skin reactions are recorded, and results from different studies show great variations regarding sensitivity, specificity, and positive predictive value [61,62,63,64]. However, there might be variations in reagents, procedure and interpretation of skin signs, limiting the accuracy of the test. One study suggested that development of erythema plus papules and/or vesicles in the skin was of high diagnostic value in children with CMA-related gastrointestinal symptoms [61]. APTs were also suggested to predict tolerance induction in non-IgE-mediated cow’s milk allergic children [65].

### 3.3. Food Challenges

Oral food challenges (OFC), especially the double-blind, placebo-controlled food challenge (DBPCFC) are recommended as the gold standard to confirm CMA, to determine the threshold dose of cow’s milk allergens in individuals, and to evaluate acquired tolerance to cow’s milk proteins [2,66]. Patients receive increasing doses of cow’s milk until adverse reactions appear (positive challenge test) or after a certain amount has been administered without reactions (negative challenge test). However, oral food challenges are time consuming, expensive, and can induce severe anaphylactic reactions that require the availability of intensive care units. Patients at high risk for anaphylaxis should receive oral food challenges only in hospitals by trained specialists [67]. Adverse symptoms involve the skin, the gastrointestinal tract, the respiratory tract, cardiovascular, or neurological symptoms, which are graded according to a severity score [68]. A recent study found that the high incidence of anaphylaxis during an OFC was related to higher sIgE levels. In particular, there was a significant association observed between anaphylaxis during the OFC with cow’s milk and the sIgE levels for caseins [28]. In addition, a history of anaphylaxis to the causative food and age were reported as risk factors for having severe anaphylactic reactions [68]. The fact that the classical path of diagnosis starting with case history and stepwise provocation testing is very time consuming is the reason why alternative approaches for diagnosis are currently emerging. These alternative approaches are based on the determination of allergen-specific IgE to a comprehensive panel of allergens in combination with medical history using provocation testing for verification of clinical relevance [69]. 

### 3.4. Molecular Diagnosis

In principle, pure allergen molecules can be produced, either by purification from the natural allergen source, or as recombinant proteins. However, it might be difficult to obtain pure preparations of natural allergens which are free of contaminating allergens from the same allergen source by biochemical purification procedures. Nowadays, the availability of cDNAs of allergens by molecular cloning techniques allows the production of recombinant proteins with high purity in big amounts (Figure 1). They represent defined isoforms which is difficult, if not impossible to achieve for natural proteins like in case of the cow’s milk allergens αS1 casein and αS2 casein [7]. When used for molecular diagnosis, recombinant allergens allow the identification of the sensitizing allergen source and IgE-reactivities to cross-reactive allergens. Dependent on the expression system they can be obtained lacking cross-reactive carbohydrates, to avoid clinically irrelevant test results. Moreover, the biological activity of recombinant allergens can be studied in *in vitro* effector cell activation assays and by skin testing in allergic patients in order to identify their clinical relevance for the induction of allergic symptoms in patients.

So far, α-lactalbumin, ß-lactoglobulin, αS1-casein, αS2-casein, and κ-casein from cow’s milk were produced as recombinant proteins in *Escherichia coli*. They have been used to study their frequencies of IgE recognition and allergenic activity in cow’s milk allergic patients [20]. Furthermore, they represent the basis for the development of therapeutic and prophylactic vaccines for cow’s milk allergy.

Since diagnostic *in vitro* tests for the measurement of specific IgE, which are based on single allergens like the ImmunoCAP system, require large amounts of serum, which is often difficult to obtain from small children, multiplex allergy tests requiring only few microliters of serum have been developed. In the recent years, these protein microarrays and other multiplex techniques have been applied to the field of allergy diagnosis. This has led to the development of allergen microarrays which are either commercially available or were produced for research purpose [18,19,20,70]. Only tiny amounts of serum are required for the determination of the IgE reactivity profiles to cow’s milk allergens and a comprehensive set of other food and respiratory allergens. A study using an experimental allergen microarray containing purified natural and recombinant cow’s milk allergens suggested that increased IgE levels to cow’s milk allergens were associated with oligo-sensitization to several cow’s milk allergens [20]. Besides specific IgE levels, the number of recognized allergens seemed to contribute to a positive food challenge result suggesting that allergen microarrays could provide useful tools to diagnose symptomatic cow’s milk allergy [18,19]. Combining the allergen microarray results with basophil activation studies using recombinant cow’s milk proteins was shown to have an additional diagnostic value, as basophil activation was mainly observed in patients with severe symptoms [20].

Cow’s milk allergen-specific IgE was shown to be a prognostic marker for persistence of cow’s milk allergy [71,72]. The development of multi-allergen assays including besides cow’s milk allergens also allergen-derived peptides allowed the mapping of sequential IgE and IgG4 epitopes, indicating that recovery from CMA was associated with decreasing IgE and increasing IgG4 to cow’s milk epitopes [73]. Furthermore, greater diversity of IgE epitope recognition and higher affinity were associated with clinical phenotypes and severity of milk allergy [74,75]. The chip-based diagnosis of comprehensive IgE reactivity profiles in conjunction with medical history has been shown to be extremely efficient for the analysis of complicated cases in children with complex IgE sensitization profiles and to facilitate the selection of personalized treatment strategies [76].

## 4. Current Treatment Strategies for Cow’s Milk Allergy

### 4.1. Avoidance and Hypoallergenic Milk Formulas

Exclusive breastfeeding is currently recommended for all infants for the first 4–6 month due to many beneficial effects of breast milk. There is also evidence that breastfeeding may reduce the development of food allergies [66]. No recommendation is currently given for pregnant and breast-feeding women to avoid cow’s milk, and maternal cow’s milk avoidance was associated with the development of cow’s milk allergy in infants [66,77,78]. In this context, the presence of allergen-specific IgA and IgG antibodies in human milk were suggested as protective factors [7,77]. A recent study highlighted the role of maternal allergen-specific IgG antibodies for the prevention of allergic sensitization to these allergens, but also peptides originating from cow’s milk allergens have been detected in breast milk, however, a possible tolerogenic effect remains to be determined [79,80]. Sensitization to human milk proteins occurs rarely, and only a few cases of suspected allergy to breast milk are reported [81,82]. While standard cow’s milk formulas are fed after the age of 4 month, hydrolysates of cow’s milk protein are recommended for children, who cannot be breastfed, children at high risk, and those who have been diagnosed with cow’s milk allergy [66] (Table 2).

Depending on the degree of hydrolysis, partially or extensively hydrolyzed milk formulas are available. Partially hydrolyzed milk formulas (phMF) usually contain whey protein derived-peptides smaller than 5 kDa, they are not considered to be completely hypoallergenic but are more easily digested than whole milk proteins. In contrast, hypoallergenic products are either extensively hydrolyzed milk formulas (ehMF), composed of small peptides <1.5 kDa, or amino acid formulas, consisting of essential and nonessential amino acids, the latter are recommended for infants who do not tolerate ehMF, but are significantly more expensive [83,84]. Milk protein hydrolysates have not only been shown to avoid allergic symptoms in cow’s milk allergic children due to the destruction of IgE epitopes but might also have immunomodulating properties like the induction of T cell tolerance and the prevention of sensitization [85]. There is evidence that hydrolyzed infant formulas may have a long-lasting preventive effect on the development of allergic symptoms [86,87], though other studies do not support the effectiveness of hydrolyzed milk formulas [88]. However, the degree and method of milk protein hydrolysis may influence the preventive effect of infant formulas on the development of milk allergy. Indeed, a recent study compared different commercially available milk formulas regarding protein and peptide content, allergenic activity, and T cell responses in terms of proliferation and cytokine production [89]. Besides their varying allergenic activity, there were striking differences of the CM formulas to induce Th1, Th2, and pro-inflammatory cytokines. Interestingly, some formulas seemed to lack immuno-stimulatory peptides, as they failed to induce any T cell proliferation or the production of proinflammatory cytokines [89].

### 4.2. Substitution of CM by Non-Bovine Milk Sources

As the elimination of cow’s milk may lead to malnutrition [90,91], milk from other sources has been proposed as a substitute for cow’s milk (Table 2). However, allergens from cow, sheep, and goat show high cross-reactivity due to their sequence homology, while mare’s, donkey’s and camel milk are better tolerated by children with CMA. [6,23,92,93]. Selective hypersensitivity to goat milk and sheep milk is rare but several cases have been reported. The degree of phosphorylation of caseins was suggested as an explanation for the differences in allergenicity among milk proteins from cow, sheep, and goat [94,95]. The use of soy-based formulas is not recommended due to the presence of phytoestrogens [96]. In addition, cross-reactivity between soy and cow’s milk proteins was reported in some patients [25,97]. Rice protein formulas represent another plant-based alternative to cow’s milk, as they lack cross-reactivity and do not contain phytoestrogens [98]. Interestingly, a gene knockout cow was recently generated which produced ß-lactoglobulin-free milk. [99]

### 4.3. Pre- and Probiotics

Prebiotics are nondigestible substances providing a beneficial physiologic effect for the host by stimulation of growth or activity of a limited number of indigenous bacteria, while probiotics represent live microorganisms, which should confer a health benefit to the host upon administration [66] (Table 2). They are currently not recommended as a dietary supplement in cow’s milk allergic patients as there is little evidence for a beneficial effect [66]. A recent meta-analysis of clinical studies investigating probiotics as treatment for food allergies in children provided some evidence that probiotics can improve symptoms of CMA but there was no evidence that they induce tolerance to cow’s milk allergens. The effects seem to depend on the administered bacterial strains, showing a beneficial effect of *Lactobacillus rhamnosus* GG [100,101]. In a recent study performed in 10-month old children the daily application of *Lactobacillus rhamnosus* and *Bifidobacterium animalis* subsp *lactis* reduced the incidence of eczema in the treated group, though the development of allergic sensitization did not differ from the placebo group [102]. Both bacterial strains reduced the risk of eczema in children with a genetic predisposition to eczema due to single nucleotide polymorphisms to TLRs. Perinatal consumption of *L. rhamnosus* GG was investigated in 303 mothers and significantly reduced the development of allergic disease in infants [103], however, maternal supplementation with *Lactobacillus rhamnosus* HN001 was shown to have no effect on infant eczema development in another study [104]. Likewise, the addition of prebiotics to a partially hydrolyzed whey formula did not prevent eczema in the first year of life, although the supplementation of the milk formula with nondigestible oligosaccharides modulated the gut microbiota closer to that of breast-fed infants. [105,106].

### 4.4. Current Strategies for Allergen-Specific Immunotherapy of Cow’s Milk Allergy

#### 4.4.1. Oral Immunotherapy (OIT)

Oral immunotherapy of cow’s milk allergic patients comprises the repeated consumption of milk allergens at regular intervals aiming to reduce the sensitivity to cow’s milk allergens during treatment, referred to as desensitization, and to induce a state of sustained unresponsiveness to the allergen after discontinuation of therapy, also known as clinical tolerance [107] (Table 2). Although standardized protocols have not been established, OIT is generally performed in 3 steps, including an escalation phase, a build-up phase, and a maintenance phase. It is currently recommended for persistent cow’s milk allergy for children from around 4–5 years of age but has recently been performed even in children less than 12 month [108]. However, it is not suited for a broad application, as it should be undertaken in clinical centers by experienced clinicians due to the risk of adverse reactions [107].

Several controlled clinical trials demonstrated the efficacy of CM OIT in cow’s milk allergic children [109,110]. They found an increase of the tolerated threshold dose, and decreased cow’s milk allergen-specific IgE levels and skin reactions in the treated groups [111,112,113,114,115]. One study reported a detailed analysis of IgE and IgG4 antibody responses to cow’s milk allergens and allergen-derived peptides before and after milk OIT. Results indicated that patients who achieved clinical tolerance had a lower and less diverse epitope-specific IgE response. After milk OIT the amount of epitope-specific IgG4 increased in contrast to epitope-specific IgE [116]. This is in line with results from a recent study suggesting that baseline sIgE levels to CM allergens were predictive for the success and safety of milk OIT [117]. However, little is known about the long-term outcomes of milk OIT, and it has been suggested that the protective effect of milk OIT requires a continuous consumption of milk [118]. Though, follow-up studies over an extended period of time for larger numbers of patients would be required [119] 

A major limitation of milk OIT is the frequent occurrence of adverse reactions. A recent study focusing on adverse events during milk OIT highlighted the inherent risks of OIT compared to allergen avoidance and identified higher IgE levels for α-lactalbumin and casein at baseline as risk factors for anaphylactic reactions during OIT [120]. 

Several approaches have been developed recently to reduce the occurrence of adverse reactions during milk OIT. Combining OIT and treatment with the monoclonal anti-IgE antibody omalizumab allowed an acceleration of the build-up phase and the reduction of the frequency and severity of side-effects in cow’s milk allergic children, but varying outcomes of efficacy were observed [121,122,123,124,125,126]. However, the high costs of this treatment have to be considered. The administration of baked milk, which has been heated at 180 °C for 30 min, has been suggested as a hypoallergenic alternative in OIT, and the consumption of baked milk products may have a beneficial effect on tolerance induction to raw cow’s milk [127]. However, severe adverse events were also observed in response to baked milk [128,129]. Hydrolyzed milk formulas, which possess lower allergenic activity than cow’s milk proteins, were also tested in OIT of cow’s milk allergic children to reduce adverse reactions during the treatment [130].

Several immunological changes that occur in the course of OIT have been described, as a decrease of allergen-specific IgE and the induction of allergen-specific IgG4 antibodies, a decline in antigen-specific Th2 cells, and the induction of regulatory T cells [126].

#### 4.4.2. Sublingual Immunotherapy (SLIT)

During OIT, the allergen is immediately swallowed, and during SLIT, it is held under the tongue for a period of time (Table 2). Sublingual immunotherapy for the treatment of CM allergic children was so far reported in only two studies. Eight children with CMA received SLIT with increasing doses of CM for 6 months. Six children who completed the study showed an increase in the eliciting dose upon challenge [131]. Keet et al. compared a SLIT protocol to OIT and combined SLIT/OIT treatment in 30 cow’s milk allergic children. Though OIT induced more severe adverse reactions, it was more efficacious for desensitization to CM than SLIT alone [132].

**Table 2:** Current strategies for the treatment of cow’s milk allergy.

Intervention	Administration Route	Procedure	Aim of Intervention	Ref.
Avoidance of CM proteins	Oral	Introduction of ehMF or aaMF into diet; Substitution of CM by non-bovine milk	Reduction of allergic symptoms due to the lack of CM-specific epitopes	[23,86,87,88,89,90,91,92,93,94,95]
OIT	Oral	Application of increasing amounts of CM	Desensitization; Clinical tolerance to CM allergens	[108,109,110,111,112,113,114,115,116,117,118,119,120,121,122,123,124,125,126,127,128,129,130]
SLIT	Sublingual	Application of increasing amounts of CM	Desensitization; Clinical tolerance to CM allergens	[131,132]
EPIT	Epicutaneous	Delivery of CM proteins via patch application	Desensitization; Clinical tolerance to CM allergens	[133,134,135,136,137,138]
Pre/ Probiotics	Oral	Given alone or in combination with hydrolyzed milk formulas	Immunomodulation	[100,101,102,103,104,105,106]

CM: cow’s milk; OIT: oral immunotherapy; SLIT: sublingual immunotherapy; EPIT: epicutaneous immunotherapy; ehMF: extensively hydrolyzed milk formulas; aaMF: amino acid milk formulas;

#### 4.4.3. Epicutaneous Immunotherapy (EPIT)

As an alternative application route of allergens for allergen-specific immunotherapy AIT, epicutaneous immunotherapy (EPIT) has been investigated for the treatment of food allergy [133,134] (Table 2). The first EPIT study in children with CMA was reported in 2010 [135]. Delivery of skimmed cow’s milk powder to the epidermis via a patch was tested in 19 children with CMA in a DBPC study. The treatment was well tolerated, adverse events were most frequently local skin reactions, and there was no incidence of systemic anaphylaxis. Though, no statistically significant improvement of the cumulative tolerated dose between the placebo and actively treated group was detected. A phase 1/2 DBPC trial (ClinicalTrials.gov identifier NCT02223182), which evaluated the safety and efficacy of a cow’s milk protein containing patch at a dose of 150, 300, or 500 µg in a larger number of milk allergic children, was recently completed, but results have not been published yet. An abstract reports that, besides mild or moderate local skin reactions, no serious adverse events occurred [136]. EPIT was also tested for the treatment of peanut allergy and for inhalant allergens using different doses and duration of application [133,137]. In addition, allergens were applied to either intact, tape-stripped, or scarified skin, which might affect the penetration of the allergens into the dermis and the activation of keratinocytes [138], indicating that more development work will be necessary for a broad application. It should be also considered that epicutaneous allergen application was found not to induce relevant production of allergen-specific IgG which is important for the success of allergen-specific immunotherapy [139,140].

## 5. Emerging Strategies for the Treatment and Prevention of Cow’s Milk Allergy

### 5.1. IgE-Targeting Therapies

Cow’s milk allergic patients are often polysensitized and exhibit elevated total IgE levels. It therefore does not come as a surprise that IgE-targeting therapies have been considered for cow’s milk allergy. In this context, a recent case report should be mentioned which described the use of IgE immunoadsorption preceding treatment with anti-IgE antibody due to extremely high IgE levels in a child with milk allergy, resulting in an increase of tolerance threshold to milk [141]. In fact, columns for selective IgE immunoadsorption have been developed and were shown to selectively deplete IgE by a few apheresis cycles thus preparing patients with very high total IgE levels for Omalizumab treatment [142,143]. In fact, beneficial effects of Omalizumab treatment on symptoms of food allergy have been recently reported for patients suffering from asthma and food allergy in a real-life study [144].

### 5.2. Subcutaneous Immunotherapy with Recombinant Hypoallergens

Although subcutaneous immunotherapy has been performed in patients suffering from respiratory allergies for more than 100 years and represents the most effective form of AIT, there is still no approved SCIT vaccine for the treatment of food allergy [1,145]. However, currently new innovative technologies for SCIT are emerging which are based on recombinant allergens and recombinant hypoallergenic allergen derivatives [140,146]. Based on the sequences of the major allergens from different allergen sources, recombinant and synthetic vaccines have been developed and already tested in clinical trials. More recently, B cell epitope-derived peptides lacking allergen-specific T cell epitopes were fused to a non-allergenic carrier and produced for the treatment of grass pollen allergy [147,148]. The latter two approaches aim at the induction of a blocking IgG antibody responses and eliminating immediate and late phase side effects. The grass pollen allergy vaccine BM32 which is based on recombinant hypoallergenic derivatives of the major grass pollen allergens has been tested now in several phase 2 clinical trials [149,150,151,152]. The studies conducted with the B cell epitope-based grass pollen allergy vaccine BM32 as well as a recent study in which cat allergic patients were treated by passive vaccination with allergen-specific monoclonal IgG antibodies highlight the important role of allergen-specific IgG-blocking antibodies for successful AIT [153]. In principle, both strategies could be also applied for treatment of food allergy [1]. A hypoallergenic derivative of the major fish allergen, parvalbumin, was developed by mutating the calcium-binding sites resulting in a loss of IgE reactivity but retained parvalbumin-specific T cell epitopes in the recombinant mutant [154]. The molecule was formulated as an aluminum hydroxide-adjuvanted vaccine for subcutaneous injection immunotherapy. It was evaluated in two phase 2 clinical trials and showed an excellent safety profile as well as the induction of parvalbumin-specific IgG antibody responses in vaccinated fish allergic patients [155,156]. Likewise, it should be feasible, that hypoallergens or B cell epitope-based recombinant vaccines derived from the major cow’s milk allergens can be developed for vaccination against cow’s milk allergy [7].

### 5.3. Strategies for the Prevention of Milk Allergy

**Figure 2:**
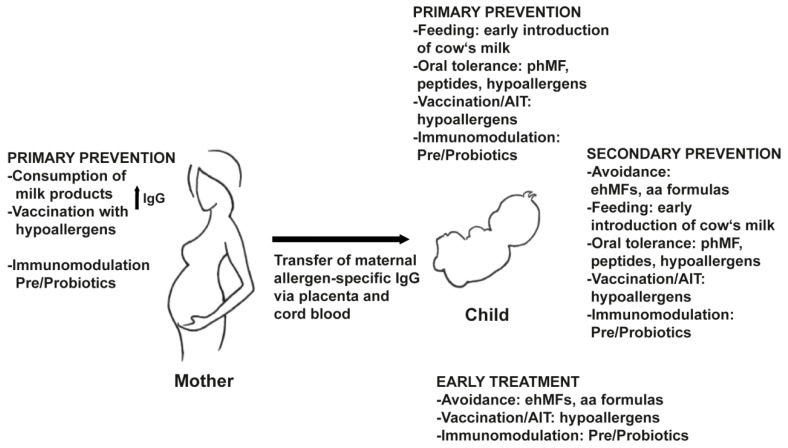
Primary, secondary prevention, and early treatment of cow’s milk allergy. Primary prevention strategies can be applied to the mother and the child to avoid allergic sensitization to cow’s milk allergens. The progression of the disease in sensitized children, or those who have already developed allergic symptoms, is inhibited by secondary prevention strategies or early treatment.

In recent years, the analysis of sensitization profiles in large birth cohorts by molecular diagnostic tests as well as prevention trials in children at risk for developing food allergy have increased our knowledge regarding the development of IgE sensitization in childhood and provided directions for early prevention measures. As depicted in Figure 2, various intervention strategies could be applied and/or developed. While primary prevention strategies aim preventing the IgE sensitization to cow’s milk allergens very early in life, secondary prevention intends to stop the transition from clinically silent IgE sensitization to symptoms of allergy and/or the progression of mild to severe symptoms. Several early treatment strategies are suggested for children with established milk allergy. 

Within the EU-funded research project MeDALL, allergen-specific IgE responses in infancy were investigated based on an allergen microarray containing >160 allergens in large European birth cohorts [4,5]. It was shown that the evolution of IgE sensitization to food and respiratory allergens starts during the first few years of life with the recognition of major allergens [157,158,159,160,161]. Several factors, like allergen-specific IgE levels, the number of recognized allergens from an allergen source and cross-reactive allergens detected early in life seem to be predictive for the onset of allergic symptoms [157,159]. IgE sensitization profiles were shown to remain stable in adulthood, suggesting that the first years of life represent a window of opportunity for early interventions [162,163]. A very recent study investigating the effects of allergen-specific IgG responses in mothers on the development of IgE sensitization in the off-springs indicated that maternal allergen-specific IgG transmitted from the mothers to the children may protect against allergic sensitization [79]. IgG reactivity to microarrayed allergens was determined during pregnancy, in cord blood samples, in breastmilk, and in children in the first years of life. It was shown that allergen-specific IgG reactivity profiles were highly correlated in mothers, cord blood, and breast milk and that maternal IgG persisted up to 6 months in children. Most importantly, children from mothers with specific plasma IgG levels >30 ISU against an allergen did not develop IgE sensitization against that allergen at 5 years of age, suggesting a highly protective effect of maternal IgG on allergic sensitization. The finding that allergen-specific IgG from mothers might prevent allergic sensitization in the offspring has already been shown in several murine studies. Protection could either occur by neutralization of the antigen by specific IgG or by uptake of allergen-IgG immune complexes via Fcgamma receptors on immune cells mediating tolerogenic effects [164]. Accordingly, primary prevention of sensitization to cow’s milk allergens in children might be initiated either by milk consumption of the mother to keep allergen-specific IgG levels high or by vaccinating the mother with hypoallergenic derivatives of cow’s milk allergens (Figure 2). The feasibility of vaccinating healthy individuals with hypoallergenic molecules was recently demonstrated in a clinical study, showing that no allergic sensitization, but the induction of an allergen-specific IgG response developed in the treated individuals [165]. These results are in agreement with previous observations suggesting that AIT treatment of pregnant women could inhibit allergic sensitization in the offspring [166]. Transfer of maternal IgG could either occur via the placenta, cord blood, and less likely by breast milk. In principle, children could receive preventive vaccines also as a prophylactic treatment in the sense of primary prevention if given before sensitization took place (Figure 2). B cell epitope-based vaccines derived from the major cow’s milk allergens might represent good candidates, as these vaccines are designed to lack allergen-specific T cell and IgE epitopes and direct the induced allergen-specific IgG responses to IgE binding sites without inducing IgE responses [151]. Likewise, passive immunization may be considered for primary prevention (Figure 2). It is known from mouse studies that the administration of allergen-specific IgG antibodies protected from subsequent allergic sensitization to this allergen [167]. However, the time window for this intervention would have to be determined in humans. 

The importance of starting a preventive treatment of food allergy at a very early timepoint in children was recently demonstrated in a clinical study investigating the prevention of peanut allergy. It was shown that the early introduction of peanut in high risk infants between 4–11 months of age significantly decreased the frequency of developing peanut allergy [168]. However, this effect could not be seen in another trial introducing six allergenic foods including cow’s milk in infants recruited from a general population [169]. At present it is not completely clear whether this intervention is an early form of OIT inducing blocking IgG or based on the induction of immunological tolerance. One also has to bear in mind that the early consumption of cow’s milk allergens as a primary or secondary prevention strategy may induce allergic sensitization to cow’s milk (Figure 2).

The feeding of hydrolyzed milk formulas may be an alternative because they are hypoallergenic and those formulas which contain intact cow’s milk allergen peptides (i.e.; partially hydrolyzed formulas) may have the capacity to induce T cell tolerance (Figure 1 and Figure 2). In fact, some cohort studies in children have indicated that hydrolyzed milk formulas may indeed suppress the development of allergic symptoms, but other studies did not confirm these results [86,87,170,171]. This might be explained by the heterogeneity of different hydrolyzed formulas [89] and the presence or absence of peptides critical for tolerance induction. As a consequence, the tolerogenic potential in hydrolyzed infant formulas is currently under investigation [172,173,174]. However, the presence of relevant T cell epitopes in these preparations depends on the use of enzymes for degradation of cow’s milk proteins. Instead, it may be preferable to produce mixtures of defined synthetic peptides comprising the cow’s milk allergen-derived T cell epitopes which could be administered for oral tolerance induction. In experimental animal models, oral tolerance induction has been shown to potently suppress allergic sensitization [175]. With the availability of recombinant allergens and synthetic allergen peptides, the doors seem to be open now for safe and robust oral tolerance induction for food as well as respiratory allergies as primary prevention [176]. 

## 6. Conclusions

Cow’s milk allergy is rare but important because it represents one of the first forms of allergy affecting children and may induce severe and life-threatening allergic reactions. Therefore, there is a need for safe forms of diagnosis, treatment and prevention. Molecular allergy diagnosis performed with recombinant allergen molecules offers an attractive alternative possibility for diagnosis in addition to oral provocation testing which may be hazardous and time consuming. Based on the detailed knowledge of the disease-eliciting allergen sources, new forms of innovative molecular AIT treatments and strategies for primary and secondary prevention of cow’s milk allergy appear on the horizon. They are based on AIT with recombinant hypoallergenic allergen molecules and tolerance induction with synthetic allergen derived peptides.
